# Understanding cancer survivors’ information needs and information-seeking behaviors for complementary and alternative medicine from short- to long-term survival: a mixed-methods study

**DOI:** 10.5195/jmla.2018.200

**Published:** 2018-01-02

**Authors:** Lou Ann Scarton, Guilherme Del Fiol, Ingrid Oakley-Girvan, Bryan Gibson, Robert Logan, T. Elizabeth Workman

## Abstract

**Objective:**

The research examined complementary and alternative medicine (CAM) information-seeking behaviors and preferences from short- to long-term cancer survival, including goals, motivations, and information sources.

**Methods:**

A mixed-methods approach was used with cancer survivors from the “Assessment of Patients’ Experience with Cancer Care” 2004 cohort. Data collection included a mail survey and phone interviews using the critical incident technique (CIT).

**Results:**

Seventy survivors from the 2004 study responded to the survey, and eight participated in the CIT interviews. Quantitative results showed that CAM usage did not change significantly between 2004 and 2015. The following themes emerged from the CIT: families’ and friends’ provision of the initial introduction to a CAM, use of CAM to manage the emotional and psychological impact of cancer, utilization of trained CAM practitioners, and online resources as a prominent source for CAM information. The majority of participants expressed an interest in an online information-sharing portal for CAM.

**Conclusion:**

Patients continue to use CAM well into long-term cancer survivorship. Finding trustworthy sources for information on CAM presents many challenges such as reliability of source, conflicting information on efficacy, and unknown interactions with conventional medications. Study participants expressed interest in an online portal to meet these needs through patient testimonials and linkage of claims to the scientific literature. Such a portal could also aid medical librarians and clinicians in locating and evaluating CAM information on behalf of patients.

## INTRODUCTION

Complementary and alternative medicine (CAM) refers to health care systems and products that are not commonly used in conventional medicine [1]. CAM includes many modalities such as yoga, chiropractic, special diets, meditation, and biologically based therapies such as dietary supplements. The use of CAM therapies by cancer patients is well documented, with current usage rates as high as 50% in the United States [2–5]. Studies have suggested positive effects from the use of CAM in the management of the psychological and emotional impact of cancer [6, 7].

When faced with a cancer diagnosis, many patients seek further information on CAM as a means of understanding their options, for a sense of hope and control, and for alternative options when the prognosis is poor [8, 9]. In this process, patients often become frustrated with what they find, particularly when they receive conflicting messages pertaining to the efficacy of CAM treatments [8, 10, 11]. Authoritative information resources and tools are needed to help patients identify and understand CAM options.

In the absence of such resources, patients often rely on anecdotal evidence, or “social proof.” Social proof is a consumer behavior where people will conform to the action of others. For example, in online shopping, social proof is often found in product reviews. Similarly, in CAM information-seeking, social proof could be found through patient testimonials [12]. Attitudes toward social proof are suggested in a recent study of CAM information seeking. One participant in that study stated, “I just kind of figured it would work well because it was a suggestion that I had heard from so many other people” [12].

To design CAM resources, it is necessary to understand the information-seeking behaviors of CAM consumers. Prior studies have focused on cross-sectional quantitative end points without considering long-term trends. One study examined the information-seeking roles of patients and caregivers [13]. The main source for CAM information was anecdotal through family and friends, with female family members playing a major role in steering male family members toward CAM use. This is not surprising, as studies have shown that women make up to 80% of the health care decisions for their families in the United States [14]. A Canadian study suggested that in many cases, patients would prefer to receive CAM information from trusted resources, such as their oncologists, but physicians were often not equipped to provide the necessary information [15].

Librarians have a professional interest in CAM information resources [16], which in part is fueled by patron requests for CAM-oriented information. In 2004, Gillaspy noted a sustained interest among consumers for information on CAM therapies [17]. In a survey of librarians, participants recorded an average comfort-level score of 3.2 (on a scale of 1–5) in responding to CAM-related information requests [18]. Professional resources supporting CAM information provision include databases [19] and website reviews [20], a study of student attitudes [21], other research published in library-focused journals, and a Medical Library Association CAM Special Interest Group. A Google search using the phrase “libguide complementary and alternative medicine” revealed that several libraries, in particular academic libraries, did provide some online guidance in locating CAM information. The outcomes of this study can further assist librarians by helping them better understand consumers’ CAM information needs and information-seeking behavior and the types of resources that can serve them.

The goal of the present study was to investigate CAM information needs and information-seeking behaviors among a cohort of long-term cancer survivors. The authors employed a mixed-method approach that consisted of a survey and in-depth interviews with Flanagan’s critical incident technique (CIT). The CIT is useful for addressing detailed questions around individuals’ motivations and decision making by eliciting in-depth stories of events. The CIT accomplishes these goals by helping the interviewee recall significant experiences that were factors in making a health care decision [19, 20]. Specifically, we looked at whether CAM use changed over time, what patients’ goals for CAM use were, where they found information, how they evaluated that information, and what their preferences were for a hypothetical CAM portal.

## METHODS

### Theoretical framework

Our study was guided by a theoretical framework composed of two theories: “Uses and Gratification Theory” (UGT) [21] and the “Health Belief Model” (HBM) [22]. These theories provided a template to guide research questions, questionnaire design, interview questions, and data analysis.

The UGT suggests that consumers seek specific mass media because they fulfill some utilitarian (uses) and emotional (gratifications) needs. The UGT provides a framework to assess the underlying motivations for consumers to seek information via the Internet or other mass media. This theory proposes that people utilize media that fulfill their needs, which fosters gratification derived from information seeking [23]. All categories of the UGT needs were applicable in our study: (1) cognitive: the desire to be informed and educated; (2) affective: satisfaction of emotional needs, such as by commiserating with another cancer patient; (3) personal integrative: self-esteem needs are met by gaining credibility such as supporting and advising other patients; (4) social integrative: the need to socialize, share experiences, and gain support from others; and (5) tension free: the use of mass media to escape from perceived reality. The UGT guided the investigation of the types of resources that would be most valuable to patients in their information-seeking processes.

The HBM examines how perceptions can be used to explain certain health behaviors [22]. Several constructs that were derived from this theory guided the investigation of the various events that triggered CAM information seeking and use. These constructs included (1) perceived threat: belief of the chances of succumbing to their disease or treatment side-effects; (2) perceived benefit: the belief in the efficacy of a treatment; and (3) perceived barriers: concerns about the impediments to successful treatment. For example, an individual’s perceived threat could motivate a need to understand his or her options to respond to clinical events such as initial diagnosis, recurrence, and adverse effects of treatments. The construct of perceived benefit may play a role in the decision to use CAM, especially when anecdotal evidence suggests that other patients have experienced positive outcomes in the use of CAM.

### Participants

Participants were recruited from those enrolled in the 2004 “Assessment of Patients’ Experience with Cancer Care” (APECC) study. APECC was population-based study that used telephone screening and mailed questionnaires to enroll 623 cancer survivors to assess their experiences with follow-up care [24]. Therefore, this cohort provided the opportunity to examine information-seeking patterns over a long-term cancer management experience.

In November 2014, 82% (510/623) of the original APECC study participants were alive. The authors sent study surveys to all these individuals and asked if they were interested in participating in the CIT interviews. Packets were mailed to potential participants between January and May of 2015.

The study was approved by University of Utah Institutional Review Board (IRB) #00068256 and the California Committee for the Protection of Human Subjects (CPHS) IRB #13-10-1383.

### Study design

Our mixed-methods approach consisted of a survey followed by CIT phone interviews of a subset of respondents. This approach combines the power of numbers and the power of stories [25, 26]. A convergent design was used where the quantitative and qualitative data collections were done sequentially, and the results were integrated after data analysis. The qualitative results provided a more detailed understanding of both significant and nonsignificant results reported by the survey.

### Survey

The CAM section from the original APECC survey was utilized in the current study to compare CAM usage over time (1994 to 2015). Additional sections were added to assess Internet use, details on information sources for CAM, and interest in various functionalities possible in an online information sharing portal. Some of the new questions were formed based on the UGT and HBM (Table 1). The complete survey and phone interview script are available in online supplemental Appendixes A and B.

**Table 1:** Theory, constructs, and sample survey questions

Theory	Construct	Sample survey question
Uses and Gratification Theory (UGT)	Cognitive	If we designed an online tool for cancer survivors to share CAM information such as CAMs used, good and bad outcomes, and the ability for patients with the same cancer to speak directly with one another, how useful would you find this tool? Would this satisfy your information needs?
	Affective	
	Social integrative	
	Personal integrative	
	Tension free	
		Please rate each portal function by interest:
	Affective	Find others with similar cancer
	Social integrative	Read testimonies of patients with similar cancer
	Personal integrative	Participate in open discussions about CAM use
	Cognitive	Generate reports for shared decision making
Health Belief Model (HBM)	Perceived benefit	What are the major reasons you used any of these [CAM] therapies?
		For which of these CAM therapies did you consult a CAM practitioner (e.g., chiropractor, naturopathic doctor)?
	Perceived barriers	Did you discuss your use of these CAM therapies with your follow-up practitioner?

### Phone interviews

The CIT [19] was used to elicit in-depth stories related to CAM information seeking that patients considered to be critical in their cancer management. A critical incident is any event in a cancer trajectory that motivates a need to search for alternatives. This technique is also tied to the HBM, in particular via the construct of perceived threat or perceived severity as well as all categories of the UGT (Table 2).

**Table 2:** Theories, constructs, and sample phone interview questions

Theory	Construct or category	Sample phone interview question
UGT	Cognitive	Consider the tools available in social networking today such as communicating person-to-person, blogging, sharing links, and sending attachments, etc. What features of a site like this would you find essential for meeting your CAM information needs?
	Affective	
	Personal integrative	
	Social integrative	
	Tension free	
HBM	Perceived threat	What caused you to seek out information on this particular product?
	Perceived severity	
	Perceived benefit	
UGT	Cognitive	How did you assess the effectiveness and safety of a CAM?
UGT	Tension free	Did you have any frustrations or concerns with the information-seeking process?

The CIT interviews consisted of five steps: (1) incident identification, (2) incident overview, (3) timeline, (4) deepening, and (5) what-if scenarios. Incident identification asked the participants to recall a specific CAM therapy that they believed was salient in meeting their wellness goals at the point when CAM was first considered. Next, participants provided an overview of their information-seeking experience. After the overview, a timeline was created, and major events such as diagnosis, recurrence, and cancer progression were identified as potential triggering events.

The description of a cancer timeline was intended to help participants recall specific events that might have caused them to seek CAM options [27]. By defining the timeline from diagnosis to the time of the interview (i.e., into long-term survivorship), the interviewees were better able to recall specific behaviors at the various points in the trajectory. The timeline was followed by a deepening phase, in which participants were asked to provide details on each event of the timeline. Participants also were asked about details such as how they were initially introduced to a CAM, how they evaluated its effectiveness, and what, if any, frustrations or concerns they experienced with the information-seeking process. The interview ended with the discussion of “what-if” scenarios related to a hypothetical CAM information-sharing portal in order to identify which functionalities (e.g., forums, testimonials) would be useful, as well as any concerns about the use of such a tool.

### Data analysis

#### Quantitative analysis

Descriptive statistics were calculated for demographics and CAM use for 2015 respondents and nonrespondents. Chi-square (McNemar’s test) was used to determine if there were significant differences where subjects were paired, and Pearson chi-square was used for unpaired subjects. Fisher’s exact test was used for unpaired groups that did not meet the assumptions of chi-square tests. Finally, Spearman’s rank correlation was used to determine if there was an association between participant demographics and interest in the functionalities available in an online CAM portal. Statistical analyses were done using Stata 14.0 and RStudio 0.99.447.

#### Qualitative analysis

The CIT interviews were recorded, transcribed, and independently analyzed by two coders using thematic analysis [28–30]. Each transcript was coded and analyzed using inductive coding and constant comparison. Emergent codes were independently entered into a codebook in Atlas.ti, and constant comparison was used to compare early codes with those from subsequent interviews. Throughout the process, new codes were identified and prior codes were refined and organized into higher level concepts, eventually leading to salient themes.

## RESULTS

### Overview

Seventy subjects (13.7%) responded to the survey. Four surveys were returned uncompleted, and 42 were returned undeliverable. Twenty-two (31.4%) of the 70 subjects returning the survey also returned a signed consent form for the phone interview. Of these, 8 (11.4%) were interviewed, 2 did not answer or return the calls, and the remaining 12 either did not seek CAM information or exclusively turned to prayer.

### Demographics

Participant demographics are summarized in Table 3. The mean age of the 2015 study respondents was 70.1 years, and the mean age of nonrespondents was 73.1 years. Respondents were more educated than nonrespondents, with 90% having at least some college as opposed to 80.1% of nonrespondents. The 2015 respondents also had higher income levels than nonrespondents.

**Table 3:** 2015 Demographics of respondents and nonrespondents

Demographic	2015 Respondents		2015 Nonrespondents		*p*-value
Age (Years)					
Younger than 50	3	(4.3%)	27	(6.1%)	
50–64	16	(22.9%)	85	(19.3%)	
65–74	27	(38.6%)	126	(28.6%)	
75 or older	24	(34.3%)	202	(45.9%)	
Age total	70	(100.0%)	440	(100.0%)	0.209
Education level					
High school or less	7	(10.0%)	88	(20.0%)	
Some college/vocational school	15	(21.4%)	145	(33.0%)	
College graduate	20	(28.6%)	87	(19.8%)	
Some graduate school/graduate	28	(40.0%)	120	(27.3%)	
Education total	70	(100.0%)	440	(100.0%)	0.009
Gender					
Male	52	(74.3%)	236	(53.6%)	
Female	18	(25.7%)	204	(46.4%)	
Gender total	70	(100.0%)	440	(100.0%)	0.001
Race					
Hispanic	6	(8.6%)	34	(7.7%)	
White	56	(80.0%)	323	(73.4%)	
Asian	6	(8.6%)	58	(13.2%)	
Other	2	(2.9%)	25	(5.7%)	
Race total	70	(100.0%)	440	(100.0%)	0.57
Income level					
Less than $20,000	3	(4.5%)	52	(12.8%)	
$20,000–$39,999	2	(3.0%)	62	(15.2%)	
$40,000–$59,999	12	(18.2%)	60	(14.7%)	
$60,000–$74,999	5	(7.6%)	62	(15.2%)	
$75,000–$99,999	14	(21.2%)	58	(14.3%)	
$100,000–$119,999	12	(18.2%)	37	(9.1%)	
$120,00 or more	18	(27.3%)	76	(18.7%)	
Income total	66	(100.0%)	407	(100.0%)	0.001

### Quantitative results

#### Complementary and alternative medicine (CAM) use

We examined the overall use of CAM, changes in the specific CAM modalities used, and use of CAM practitioners by respondents between 2004 and 2015. Although there was no significant change in use of the various CAM modalities, there was a significant increase in use of CAM practitioners. Out of the seventy respondents to the 2015 survey, sixty-one also provided this information in the 2004 study (Table 4).

**Table 4:** CAM usage patterns for those reporting usage in both 2004 and 2015 surveys (n=61)

CAM	2004		2015		*p*-value
	
	n	(%)	n	(%)	
Special diets	16	(26.2%)	18	(29.5%)	0.802
Movement therapies	10	(16.4%)	14	(23.0%)	0.299
Supplementation	8	(13.1%)	6	(9.8%)	0.724
Homeopathy	2	(3.3%)	1	(1.6%)	>0.99
Mind and body	10	(16.4%)	8	(13.1%)	0.752
Oriental therapies	4	(6.6%)	5	(8.2%)	>0.99
Self help	4	(6.6%)	6	(9.8%)	0.752
Psych	4	(6.6%)	6	(9.8%)	0.724
Faith healing	7	(11.5%)	4	(6.6%)	0.505
Prayer	28	(45.9%)	22	(36.1%)	0.181
Overall CAM use	37	(60.7%)	36	(59.0%)	>0.99
CAM use excluding prayer	35	(57.4%)	29	(47.5%)	0.146
Used CAM practitioners	6	(9.8%)	16	(26.2%)	0.004

#### CAM information sharing portal

**Figure 1:**
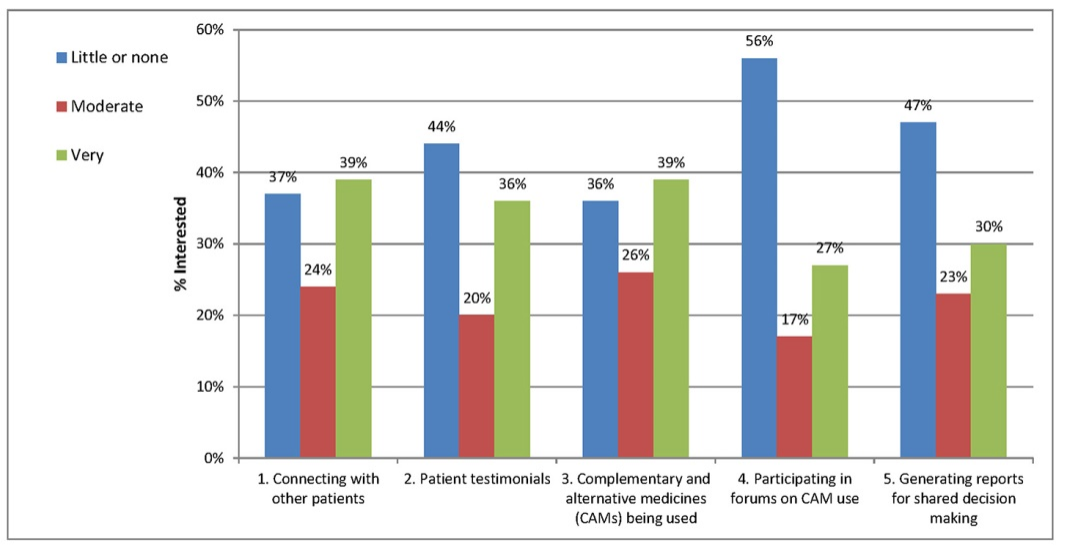
Interest in online portal functionality

Figure 1 shows participants’ interest in web portal functionality for helping meet CAM information needs. More than half of the study population indicated interest in each option, with the exception of participating in forums.

There were statistically significant associations for both gender and age with the following portal functions: connecting with other patients, testimonials, and CAMs being used. Women and younger people expressed higher interest in those CAM portal functions. Income and education were not significantly correlated with interest in CAM portal functionality (Table 5).

**Table 5:** Spearman correlations between demographics and interest in portal functionality (n=55)

	Connecting with others	Testimonials	CAMs being used	Participating in forums	Generating reports
Age	−0.419*	−0.399*	−0.439*	−0.264	−0.379*
Education	−0.034	0.022	0.109	0.002	0.068
Income	0.099	0.036	0.169	0.064	0.152
Gender	0.380*	0.341*	0.276*	0.227	0.210

55/70 completed this portion of the survey.

* *p*<0.05

### Qualitative results: critical incident interviews

#### Thematic analysis

The thematic analysis resulted in the following themes.

1. *Lack of emotional and psychological support during treatment*. The study’s participants were satisfied with their care for the direct treatment of their cancer but felt alone in dealing with the emotional and psychological impact.

You go to very dark places when you get sick. I hit my breaking point many years ago, then I started feeling like I was going to give up. I got back into the program of yoga and deep breathing…I tell people you have to build yourself a little tool chest. Those tools can be group meetings, mentoring calls, yoga, it can be this, all these little things that keep you busy promoting your wellness. I really like that metaphor of my little tool kit, and I pull it out when I feel like this.

2. *Utilization of trained CAM practitioners.* Participants used varying forms of CAM that required the use of trained practitioners.

I went to a Naturopath and got some of the candida in my body out and we started to clean me up from all the toxins I was under from eating the normal American diet, the wrong foods and that kind of stuff, over the years.

3. *Online resources as a prominent source for CAM information*. Participants discussed how their preferred information sources during their treatment had changed. Participants utilizing printed materials now used online resources. Participants who still relied on friends and family used the Internet for additional evidence of efficacy as well as possible interactions. A participant was asked if she were diagnosed with a serious illness today, where would she turn first for treatment options.

Online! Totally. I’m always looking at online things and trying to make connections. I’m just kind of curious because I’m a Chemist so I have a lot of curiosity about the science behind it.

4. *Friends and family as sources*. Participants indicated that their initial introduction to CAM was often via family or friends.

[A]t that point, I was kind of letting my Mom, you know, I kind of put my trust in her and let her make those decisions. She was the one that had access to information.

#### Interest in functionality for an online information-sharing portal

Study participants rated their interest in functionality that could be available through an online CAM information sharing portal. The functionality discussed includes the following.

1. *Connection with other patients*. Participants placed a strong emphasis on the need to connect with others who truly understand what they are going through.

There was a point where I told my Oncologist that I was depressed, and his answer was, “well, we’ll prescribe you some Prozac”,…but I didn’t want a drug, I didn’t want a pill, I don’t want Prozac. I wanted to just be able to communicate. I’m depressed because I don’t know what I’m going through, I don’t understand what I’m going through, there’s no one I know that can relate to what I’m going through, and it’s like everything is unknown, there’s no outlet, and that’s what I was trying to tell him.

2. *Patient testimonials*. Study participants sought to hear about what treatment approaches others have tried and the outcomes.

That’s really great. If you were diagnosed with something that gave you six months to live, and you talked to someone that said “I’ve tried this”, they said I only had six months to live, but I’m talking to you several years later, how much better is that going to make you feel, just because it increases your chance of survival.

3. *Participation in forums*. Interviewees seemed to have a more positive picture about participation in forums compared to the survey findings.

I value my anonymity, I wouldn’t want people to know who I was, but at the same time, I would like to share my story. You know, if it would be of benefit to other people, and I would like to know what other people have to say, but I don’t necessarily need to know who people are.

4. *Reports for shared decision making*. Although no strong feelings were expressed about this functionality, phone interviewees expressed confidence in their physicians. One participant emphasized she would not do anything that her physician was not aware of and approved.

Yeah, and my gut tells me he wouldn’t just say don’t do that, he would tell me why he thinks that. I’m not going to just go out and try something without talking to my doctors first, if I think it’s going to help me, or give me more energy, or boost my blood levels so they aren’t just at normal or below normal all the time, I would definitely have to talk to someone first. I would definitely look at the web site, see what’s going on, make some notes reminding me to talk to my doctors the next time I call them, hey, have you heard anything about this? I would definitely have to have the conversation with my doctors first.

## DISCUSSION

To our knowledge, this is the first study to investigate cancer survivors’ CAM information needs and information-seeking behaviors using a mixed-methods approach. This study has multiple strengths, including recruitment from an existing cohort of cancer survivors and a theory-guided, mixed-methods approach. Utilizing an existing cohort from the 2004 APECC study facilitated comparison over time with previously collected data on CAM usage and information sources.

Overall CAM usage was high (60% reported using CAM), with no significant difference between 2004 and 2015. The primary motivation for CAM use was to manage the emotional and psychological impact of cancer and its treatments, and participants were usually left to seek options from outside sources [8]. More than 50% of the surveyed long-term survivors expressed interest in an online information sharing portal for CAM. The phone interviews indicated that use of anecdotal sources is still prevalent, and study subjects go online to find evidence from the scientific literature, seek social networks for further information on the use of a CAM, and search consumer health information sites such as WebMD®.

While assessing the reasons that survivors sought CAM options, we found that the primary motivators were the threat of treatment and posttreatment recovery. These motivations were consistent with the perceived threat construct in the HBM as well as the perceived benefit, which was consistent with a similar study of patients with Lyme disease [31]. Patients’ psychological distress resulted in searches for options and increased CAM use. CAM use has been shown to provide patients with a sense of hope and control and may be motivated by multiple categories of the UGT, including cognitive, affective, and tension free.

Of the more than 50% expressing interest in an online information sharing portal for CAM, women and younger participants expressed greater interest. The role of women in information-seeking and health care decision making for family members is well documented [14, 15]. The phone interviews documented similar behaviors amongst women and men. Two of the eight interviewees indicated that their CAM usage was driven primarily by women in their lives; in one case, the mother, and in another, a wife.

Fewer than 50% of respondents expressed interest in discussion forums. The qualitative results suggested concerns over privacy. Multiple phone interviewees said they would actively participate in forums if participation was anonymous.

Similar to a previous study, we found that there was a large gap in cancer patients’ needs in both dealing with the psychological and emotional aspects of the diagnosis and treatments, as well as recovering strength and overall health after treatment. In a previous study, Kent et al. found “ notable proportion of survivors of leukemia, bladder, and colorectal cancer reported symptom bother and unmet supportive care needs months or years beyond their cancer diagnosis” [32], which might have resulted in ongoing CAM needs across the cancer continuum.

Community and medical libraries might use findings from this study to guide how they provide resources and services to patrons with CAM information needs. First, libraries can help patients to evaluate evidence-based resources through criteria such as the Health on the Net (HON) Code. Second, librarians can educate patients on how to maintain privacy and anonymity while using online forums. Finally, a library’s website can provide a starting point to assist with the aforementioned services as well as others related to CAM information. These efforts could be expanded to develop an online CAM portal guided by the results of the current study.

Several portals address some of the consumer needs exposed in this paper. Life Extension is a supplement company that links claims of efficacy to the scientific literature. Natural Medicines Comprehensive Database provides information on the efficacy of various CAM modalities, with links to supporting literature. A third resource, Patients Like Me, is the only portal that supports patient input. Overall, the results of this study could be used to improve these portals to meet consumer needs for CAM.

This study has several implications for future research. Participants in our study suggested health information portals would be particularly valuable if anecdotal claims were linked to the scientific literature as further support of the efficacy and safety of a CAM treatment. The lack of significant changes in CAM usage patterns many years into survivorship poses interesting questions for follow-up studies on why long-term survivors continue to use CAM at a high rate. Although one such study exists, it focuses on mind-body therapies and reports a decline in use in long-term survivorship, which is inconsistent with our findings [33]. A suggested future study could assess issues pertaining to cancer survivorship and what new CAMs patients use to address treatment-related issues such as radiation damage, nerve damage, and mood disorders.

This study has several limitations. First, the respondents to the current study were more highly educated and were from a higher income bracket. This might indicate higher health literacy as well as greater access to web-based resources. Second, the survey had a small response rate (13.7%), introducing selection bias. Third, the original sample was drawn from the Greater Bay Area Cancer Registry, a population-based registry that contributes to the SEER program. As documented by Arora and colleagues [24], compared with the eligible nonrespondents, respondents were more highly educated, had a higher income level, and were mostly male. Finally, the CAM information-seeking critical incidents often occurred several years ago, compromising the participants’ recall of details. Given the study’s limitations, the findings’ generalizability to the full US cancer survivor population cannot be assessed. However, the current study’s results contribute to a growing CAM literature and provide additional data to generate future research.

## Supplemental Files

Appendix A Survey

Appendix B Phone interview script
