# Supplementary material for: Understanding cancer survivors’ information needs and information-seeking behaviors for complementary and alternative medicine from short- to long-term survival: a mixed-methods study
Source: J Med Libr Assoc. 2018 Jan 2;106(1):87–97. doi: 10.5195/jmla.2018.200 (PMC5764598; doi:10.5195/jmla.2018.200)
Supplement: Appendix A [file jmla-106-87-s001.pdf]

JMLA Journal of the Medical Library Association

SC

Supplemental content to
J Med Libr Assoc. Jan;106(1):dx.doi.org/10.5195/jmla.2018.200
www.jmla.mlanet.org
© Scarton, Del Fiol, Oakley-Girvan, Gibson, Logan, Workman 2018

Understanding cancer survivors' information needs and information-seeking behaviors for complementary and alternative medicine from short- to long-term survival: a mixed-methods study

Lou Ann Scarton; Guilherme Del Fiol; Ingrid Oakley-Girvan; Bryan Gibson; Robert Logan; T. Elizabeth Workman

APPENDIX A

Survey
ID: _________________________
Conducted by: University of Utah School of Medicine
CONFIDENTIAL

Introduction

Complementary and alternative medicine (CAM) use in on the rise. Despite this, we know relatively little about where patients find CAM information, what specific questions they ask, and how they evaluate the answers to those questions. The University of Utah, in collaboration with Cancer Prevention Institute of California (CPIC), formerly known as the Northern California Cancer Center (NCCC), is conducting a questionnaire study of cancer survivors as a follow-up to the 2004 "Assessment of Patients' Experience of Cancer Care" (APECC) study. You have been selected to participate in this important research study as one of the over 600 survivors participating in the 2004 study. The main purpose of the study is to help researchers understand patients' information needs for CAM and address those needs through a CAM website open to all patients with experiences to share, as well as those simply looking for information. Through this website, patients with similar cancers can discuss what CAMs they have used and the outcome of that use, both positive and negative, in order to make a more informed decision about CAM use.

This survey booklet contains questions on several topics. These include questions about your information needs, as well as your Internet usage for satisfying your health care information needs. These questions will allow us to design a better website for patient-reported outcomes for CAM. We know of no better way to evaluate these needs other than to ask cancer survivors themselves. Hence, your input is very important to us.

There are no right or wrong answers, so you should respond by giving the answer that best describes your own needs. While we would like you to answer all the questions in this booklet; however, you may skip any question that you do not wish to answer. Even if you feel you must skip a question, your answers to other questions will still be important to us.

All of the information you provide is confidential and will be used for research purposes only. Your information will not be disclosed to your health care provider, and your participation will not affect your health care in any way. The information obtained will be analyzed as grouped data without any personal identification. When you are completing this survey, if any issues concern you about your health, please discuss these with your health care provider. You are also free to contact us by telephone or email to discuss any issues related to the survey material.

Please do not be concerned about the inconsistent identifiers used for each study question. Several are questions you answered in the original study and, therefore, have the same identifier with a "-2" suffix. Others are new questions for this study.

JMLA Journal of the Medical Library Association

SC
Supplemental content to
J Med Libr Assoc. Jan;106(1):dx.doi.org/10.5195/jmla.2018.200
www.jmla.mlanet.org
© Scarton, Del Fiol, Oakley-Girvan, Gibson, Logan, Workman 2018

If you have any questions, please contact the principal investigator.

We are very grateful for the time you are taking to complete this survey and for helping us learn how to improve the quality of health care information for cancer survivors. After completing this survey, please mail it back to us in the enclosed postage-paid envelope.

Thank you.

PLEASE READ THESE INSTRUCTIONS CAREFULLY
GENERAL INSTRUCTIONS

- Answer each question as best you can. Please do not leave any question blank.
- Put an x or a √ in the box next to your answer and erase or cross out completely if you make any changes.
  Example: 1☑ Yes : 1☒ Yes
- Please follow any instructions that direct you to the next question.
  Example: 2☑ No → GO TO A11
- If you mark an answer with a line after it, please write the specific information on the line provided.
  Example: 1☑ Other, please specify: cardiologist
- Mark only one response for each question, unless directed to "MARK ALL THAT APPLY." For those questions, please mark every response choice that applies to your situation.
- As far as possible, please try to answer all the questions in one sitting and, where asked, please record the time you started and ended this survey.

A. Study information

A1-2. Please record the time you begin answering this survey. Write the hour on the line and check the box for a.m. or p.m.: ____________

1 a.m.
2 p.m.

A2-2. Today's date:

____________ ____________ ____________
MONTH DAY YEAR

A15-2. To the best of your knowledge, are you now free of cancer (that is, at this time, you don't have cancer in any part of your body)?

1 Yes
2 No

C. Complementary and alternative therapies used in the last 12 months

Introduction

This section contains a short list of questions that you answered in the original 2004 APECC study. Our goal here is to determine if your answers have changed since the original study.

SC
Supplemental content to
J Med Libr Assoc. Jan;106(1):dx.doi.org/10.5195/jmla.2018.200
www.jmla.mlanet.org
© Scarton, Del Fiol, Oakley-Girvan, Gibson, Logan, Workman 2018

C1-2. In the **last 12 months,** did you use any of the following complementary and alternative therapies?

	**Yes**	**No**
a. Special diets such as mostly vegetarian or low fat	1	2
b. Movement or physical therapies such as yoga, tai chi, massage, chiropractic, or electromagnetic therapy	1	2
c. High dose or mega vitamins (DO NOT include 1-a-day multivitamins), nutritional supplements, or herbal remedies	1	2
d. Homeopathy	1	2
e. Mind/body therapies such as guided imagery/visualization, biofeedback, meditation, relaxation techniques, hypnosis/hypnotherapy, energy healing, therapeutic touch, or music therapy	1	2
f. Oriental therapies such as acupuncture, acupressure, qigong, or shiatsu	1	2
g. Self-help or support groups (either face-to-face or on the Internet)	1	2
h. Psychological therapy or counseling from a psychologist, psychiatrist, social worker, or any other mental health professional	1	2
i. Faith healing, laying on of hands, or any other spiritual or religious group experience	1	2
j. Personal prayer or personal spiritual healing	1	2
k. Other, please specify: ________________________________	1	2

If you DID NOT USE any of the above therapies in the LAST 12 MONTHS, please go to section K.

If you USED one or more of the above therapies in the LAST 12 MONTHS, please continue with question C2-2.

C2-2. What were the major reasons why you used any of these therapies discussed in question C1 in the last 12 months?

MARK ALL THAT APPLY

0☐ I didn't use any of these therapies in the last 12 months → GO TO SECTION K
1☐ To relieve symptoms or any treatment-related side effects (such as pain, nausea, fatigue, anxiety, depression, or other similar symptoms/side-effects)
2☐ To relieve stress
3☐ To treat my cancer
4☐ To prevent my cancer from coming back (recurrence)
5☐ To help deal with a medical condition other than cancer, please specify: ______________
6☐ To promote health and overall well-being
7☐ Not satisfied with conventional follow-up care
8☐ Wanting control
91☐ Other, please specify: ______________________________

C3-2. Overall, how helpful were any of these therapies you used in the **last 12 months** in meeting the above goals?

1☐ Not at all helpful
2☐ Somewhat helpful
3☐ Very helpful

SC
Supplemental content to
J Med Libr Assoc. Jan;106(1):dx.doi.org/10.5195/jmla.2018.200
www.jmla.mlanet.org
© Scarton, Del Fiol, Oakley-Girvan, Gibson, Logan, Workman 2018

C4-2. In the **last 12 months,** did you see any complementary and alternative therapy practitioner or doctor to receive any CAM therapies?

1☐ Yes
2☐ No → GO TO C5-2

C4-2.a. As best as you can, please tell us the specialty of all the complementary and alternative therapy practitioners or doctors you saw in the **last 12 months:**

____

C4-2.b. Overall, how would you rate the quality of care you received from the complementary and alternative therapy practitioners or doctors you saw in the last 12 months?

1☐ Poor
2☐ Fair
3☐ Good
4☐ Very good
5☐ Excellent

C4-2.c. For which CAMs listed below did you consult a CAM practitioner such as a naturopathic doctor (ND), chiropractor, or other professional for guidance.

	**Yes**	**No**
a. Special diets such as mostly vegetarian or low fat	1	2
b. Movement or physical therapies such as yoga, tai chi, massage, chiropractic, or electromagnetic therapy	1	2
c. High dose or mega vitamins (DO NOT include 1-a-day multivitamins), nutritional supplements, or herbal remedies	1	2
d. Homeopathy	1	2
e. Mind/body therapies such as guided imagery/visualization, biofeedback, meditation, relaxation techniques, hypnosis/hypnotherapy, energy healing, therapeutic touch, or music therapy	1	2
f. Oriental therapies such as acupuncture, acupressure, qigong, or shiatsu	1	2
g. Self-help or support groups (either face-to-face or on the Internet)	1	2
h. Psychological therapy or counseling from a psychologist, psychiatrist, social worker, or any other mental health professional	1	2
i. Faith healing, laying on of hands, or any other spiritual or religious group experience	1	2
j. Personal prayer or personal spiritual healing	1	2
k. Other, please specify: ____________________	1	2

JMLA Journal of the Medical Library Association
Supplemental content to
J Med Libr Assoc. Jan;106(1):dx.doi.org/10.5195/jmla.2018.200
www.jmla.mlanet.org
© Scarton, Del Fiol, Oakley-Girvan, Gibson, Logan, Workman 2018

C5-2. Did you discuss your use of any of these complementary and alternative therapies with your follow-up care doctor in the **last 12 months?**

0☐ I did not receive follow-up cancer care in the last 12 months → GO TO SECTION J
1☐ Yes
2☐ No → GO TO C6-2

C5-2.a. Which of the following best describes your follow-up care doctor's response?
Your doctor…
1☐ Encouraged you to use it
2☐ Didn't care whether you used it or not
3☐ Told you about the risks in using it
4☐ Encouraged you to stop using it
5☐ Made no comment
91☐ Other, please specify: ______________________
PLEASE GO TO SECTION J

C6-2. What were the main reasons why you didn't discuss your use of complementary and alternative therapies with your follow-up care doctor in the last 12 months?
MARK ALL THAT APPLY
1☐ Your doctor never asked
2☐ You thought that your doctor wouldn't approve
3☐ It wasn't important for you to tell your doctor
4☐ You felt your doctor might refuse to continue to be your doctor
91☐ Other, please specify ________________________________

J. Complementary and alternative medicine information seeking

Introduction

This section is intended to get you thinking specifically about a CAM that you use regularly and to share how you learned about it and what sources you would prefer for this information if they were available.

J1. Think of a complementary or alternative medicine that you currently use. Where did you find out about this CAM?

	**Did not use**	**Used infrequently**	**Used frequently**	**My primary source**
a. Family	1	2	3	4
b. Friends	1	2	3	4
c. Naturopathic doctor	1	2	3	4
d. Physician	1	2	3	4
e. Nurses	1	2	3	4
f. Other patients using CAM	1	2	3	4
g. The Internet	1	2	3	4
h. Other media such as printed materials	1	2	3	4

Supplemental content to
J Med Libr Assoc. Jan;106(1):dx.doi.org/10.5195/jmla.2018.200
www.jmla.mlanet.org
© Scarton, Del Fiol, Oakley-Girvan, Gibson, Logan, Workman 2018

J2. How useful did you find the following sources when you decided to use that CAM?

	Not useful	Somewhat useful	Quite useful	Extremely useful
a. Family	1	2	3	4
b. Friends	1	2	3	4
c. Naturopathic doctor	1	2	3	4
d. Physician	1	2	3	4
e. Nurses	1	2	3	4
f. Other patients using CAM	1	2	3	4
g. The Internet	1	2	3	4
h. Other media such as printed materials	1	2	3	4

J3. Please indicate what type(s) of information you preferred when evaluating that complementary or alternative treatment, whether it was available or not.

	Never	Sometimes	Usually	Always
a. Physician's recommendation	1	2	3	4
b. Naturopathic doctor's recommendation	1	2	3	4
c. Patient testimonials	1	2	3	4
d. Scientific evidence/studies	1	2	3	4
e. Family/friend recommendation	1	2	3	4
f. Other	1	2	3	4

J3a. If you selected "Other," what source of information did you use or would you prefer if it were available?

K. Patient CAM information sharing portal

Introduction

This section is intended to help us understand user needs in order to design an online tool to meet those CAM information needs. This section describes some of the functionality this tool may have and allows you to indicate whether this would be useful to you in making decisions about CAM use. The last two questions allow you to request specific functionality should you feel those listed below do not meet your needs.

K1. If we designed an online tool for cancer survivors to share CAM information such as CAMs used, good and bad outcomes, and the ability for patients with the same cancer to speak directly with one another, how useful would you find this tool?

1☐ Never useful
2☐ Almost never useful
3☐ Occasionally useful
4☐ Almost always useful
5☐ Always useful

JMLA Journal of the Medical Library Association
SC
Supplemental content to
J Med Libr Assoc. Jan;106(1):dx.doi.org/10.5195/jmla.2018.200
www.jmla.mlanet.org
© Scarton, Del Fiol, Oakley-Girvan, Gibson, Logan, Workman 2018

K2. If we designed a site as described above, how likely would you use it?
1☐ Extremely unlikely
2☐ Unlikely
3☐ Neutral, may explore it
4☐ Likely
5☐ Extremely likely

K3. If we designed a site as described above, how often would you use it?
1☐ Never
2☐ Almost never
3☐ Neutral, would need to see it to determine interest
4☐ Occasionally
5 Frequently

K4. The following are some examples of what may be available on the CAM site above. Please rate each function by your <u>interest.</u>

K4a. Find others with similar cancer
1☐ Not at all interested
2☐ Slightly interested
3☐ Somewhat interested
4☐ Very interested
5☐ Extremely interested

K4b. Read testimonies of patients with similar cancer
1☐ Not at all interested
2☐ Slightly interested
3☐ Somewhat interested
4☐ Very interested
5☐ Extremely interested

K4c. Find out about what kinds of CAM other similar patients are taking
1☐ Not at all interested
2☐ Slightly interested
3☐ Somewhat interested
4☐ Very interested
5☐ Extremely interested

K4d. Participate in open discussions about CAM use (blogs)
1☐ Not at all interested
2☐ Slightly interested
3☐ Somewhat interested
4☐ Very interested
5☐ Extremely interested

K4e. Ability to generate reports to share with my physician with CAM claims patients are making before making my own decision.
1☐ Not at all Interested
2☐ Slightly interested
3☐ Somewhat interested
4☐ Very interested
5☐ Extremely interested

SC
Supplemental content to
J Med Libr Assoc. Jan;106(1):dx.doi.org/10.5195/jmla.2018.200
www.jmla.mlanet.org
© Scarton, Del Fiol, Oakley-Girvan, Gibson, Logan, Workman 2018

K5. How often *would you use* each of the following options if they were available?

K5a. Find others with similar cancer
1☐ Never
2☐ Rarely
3☐ Occasionally
4☐ Almost every time
5☐ Every time

K5b. Read testimonies of patients with similar cancer
1☐ Never
2☐ Rarely
3☐ Occasionally
4☐ Almost every time
5☐ Every time

K5c. Find out about what kinds of CAM other similar patients are taking
1☐ Never
2☐ Rarely
3☐ Occasionally
4☐ Almost every time
5☐ Every time

K5d. Participate in open discussions about CAM use (blogs)
1☐ Never
2☐ Rarely
3☐ Occasionally
4☐ Almost every time
5☐ Every time

K5e. Ability to generate reports to share with my physician with CAM claims patients are making before making my own decision.
1☐ Never
2☐ Rarely
3☐ Occasionally
4☐ Almost every time
5☐ Every time

K6. Other functions you would like to see available on a CAM site

---

K7. What other tools or websites would you find helpful to meet your CAM information needs?

L. Additional comments

<u>Introduction</u>

This section is used to explore your Internet use in general. This will help us understand if Internet-based tools and social networking type solutions will meet the needs of a majority of those patients searching for CAM-related information.

JMLA Journal of the Medical Library Association

SC
Supplemental content to
J Med Libr Assoc. Jan;106(1):dx.doi.org/10.5195/jmla.2018.200
www.jmla.mlanet.org
© Scarton, Del Fiol, Oakley-Girvan, Gibson, Logan, Workman 2018

L1. How often did you use the Internet in 2004 at the time of the original APECC study?
1☐ Never
2☐ Rarely
3☐ Occasionally
4☐ A moderate amount
5☐ A great deal

L2. How often do you use the Internet now?
1☐ Never
2☐ Rarely
3☐ Occasionally
4☐ A moderate amount
5☐ A great deal

L3. Do you use social networking sites such as Facebook?
1☐ Never
2☐ Rarely
3☐ Occasionally
4☐ A moderate amount
5☐ A great deal

L4. If you use social networking, approximately how long have you been actively using it?
1☐ Do not use
2☐ < 1 year
3☐ 1–3 years
4 3–5 years
5☐ > 5 years

L5. How often do you use your social media to discuss health-related information?
1☐ Never
2☐ Rarely
3☐ Occasionally
4☐ A moderate amount
5☐ A great deal

L6. How often do you use your social media to discuss CAM?
1☐ Never
2☐ Rarely
3☐ Occasionally
4☐ A moderate amount
5☐ A great deal

L7. How often do you use your social media to discuss healthy eating and lifestyle topics?
1☐ Never
2☐ Rarely
3☐ Occasionally
4☐ A moderate amount
5☐ A great deal

Supplemental content to
J Med Libr Assoc. Jan;106(1):dx.doi.org/10.5195/jmla.2018.200
www.jmla.mlanet.org
© Scarton, Del Fiol, Oakley-Girvan, Gibson, Logan, Workman 2018

L8. What devices do you use to access the Internet (check all that apply)?
1☐ Tablet device (such as iPad, MS Surface, Samsung Galaxy, or similar)
2☐ Smart phone
3☐ Laptop computer
4☐ Desktop computer

L9. Finally, if you have any comments about this survey or would like to share any concerns or problems related to or due to your cancer that we did not cover in this survey, please feel free to do so below:

M. Perceived health competence

Introduction

This section explores how confident patients feel in their ability to locate health information, evaluate the information, and incorporate it into their wellness plans. If a large percentage of patients do not feel they can understand what they are reading and how a given treatment will improve their health, then an online tool providing this information may not be helpful.

M1. I handle myself well with respect to my health.
1☐ Strongly disagree
2☐ Disagree
3☐ Undecided
4☐ Agree
5☐ Strongly agree

M2. No matter how hard I try, my health just doesn't turn out the way I would like.
1☐ Strongly disagree
2☐ Disagree
3☐ Undecided
4☐ Agree
5☐ Strongly agree

M3. It is difficult for me to find effective solutions to the health problems that come my way.
1☐ Strongly disagree
2☐ Disagree
3☐ Undecided
4☐ Agree
5☐ Strongly agree

M4. I succeed in the projects I undertake to improve my health.
1☐ Strongly disagree
2☐ Disagree
3☐ Undecided
4☐ Agree
5☐ Strongly agree

M5. I'm generally able to accomplish my goals with respect to my health.
1☐ Strongly disagree
2☐ Disagree
3☐ Undecided
4☐ Agree
5☐ Strongly agree

M6. I find my efforts to change things I don't like about my health are ineffective.
1☐ Strongly disagree
2☐ Disagree
3☐ Undecided
4☐ Agree
5☐ Strongly agree

M7. Typically, my plans for my health don't work out well.
1☐ Strongly disagree
2☐ Disagree
3☐ Undecided
4☐ Agree
5☐ Strongly agree

M8. I am able to do things for my health as well as most other people.
1☐ Strongly disagree
2☐ Disagree
3☐ Undecided
4☐ Agree
5☐ Strongly agree

N. Integrative medicine

Introduction

This section explores how the patients feel about the availability of integrative medicine (IM) services in a clinic or hospital they are considering for their care. IM refers to the inclusion of therapies that are generally considered to be outside traditional medicine. They could include mind/body therapies such as meditation, yoga, and music therapy as well as biologically based therapies such as supplements, special diets, and cleansing.

N1. I have been very curious about integrative medicine (IM) and would like to know more about it.
1☐ Strongly disagree
2☐ Disagree
3☐ Undecided
4☐ Agree
5☐ Strongly agree

SC
Supplemental content to
J Med Libr Assoc. Jan;106(1):dx.doi.org/10.5195/jmla.2018.200
www.jmla.mlanet.org
© Scarton, Del Fiol, Oakley-Girvan, Gibson, Logan, Workman 2018

N2. I would likely choose a clinic that had expertise in IM over others clinics.
1☐ Strongly disagree
2☐ Disagree
3☐ Undecided
4☐ Agree
5☐ Strongly agree

N3. I believe that IM shows great promise in improving patient outcomes.
1☐ Strongly disagree
2☐ Disagree
3☐ Undecided
4☐ Agree
5☐ Strongly agree

N4. I believe that IM is one possible answer to rising health care costs.
1☐ Strongly disagree
2☐ Disagree
3☐ Undecided
4☐ Agree
5☐ Strongly agree

O. Personal perspective on information needs

Introduction

Many thoughts and feelings are expressed by patients in discussing their CAM information-seeking experiences. If you have spent time searching for CAM information and had difficulties, please tell us a bit about what caused the greatest anger, frustration, fear, or any other emotion you may have had.

O1. I have experienced the following emotions while trying to understand my options by searching for CAM information.

____

O2. Please note the time at which you finished the survey: ______________________
1☐ a.m.
2☐ p.m.

O3. If you answered the survey in more than one sitting, please fill in the approximate total time you spent answering all the questions in this survey: ________________________

THANK YOU for taking the time to fill out this survey.

Please return the survey in the enclosed postage-paid envelope as soon as possible.
