# Supplementary material for: Understanding cancer survivors’ information needs and information-seeking behaviors for complementary and alternative medicine from short- to long-term survival: a mixed-methods study
Source: J Med Libr Assoc. 2018 Jan 2;106(1):87–97. doi: 10.5195/jmla.2018.200 (PMC5764598; doi:10.5195/jmla.2018.200)
Supplement: Appendix B [file jmla-106-87-s002.pdf]

JMLA Journal of the Medical Library Association

Supplemental content to
J Med Libr Assoc. Jan;106[1]:dx.doi.org/10.5195/jmla.2018.200
www.jmla.mlanet.org
© Scarton, Del Fiol, Oakley-Girvan, Gibson, Logan, Workman 2018

Understanding cancer survivors' information needs and information-seeking behaviors for complementary and alternative medicine from short- to long-term survival: a mixed-methods study

Lou Ann Scarton; Guilherme Del Fiol; Ingrid Oakley-Girvan; Bryan Gibson; Robert Logan; T. Elizabeth Workman

APPENDIX B

Phone interview script

Verbal introduction and consent script

[When a person answers the phone:]
Hello, My name is [Author] and I am calling on behalf of the "Assessment of Patients' Experience with Cancer Care" [APECC] follow-up study. May I speak to [Participant name]?

Speaking to participant: Go to Intro A

Participant unavailable: Go to Intro B

Intro A

Within the past few weeks, you completed a survey form as a follow-up to the APECC study. I would like to thank you for your valuable input. You also completed the contact form to participate in the phone interview to provide a more detailed description of your complementary and alternative medicine (CAM) information-seeking experiences. Assuming you would still like to participate, is this a good time?

If yes: Continue with CAM phone interview

If no: Request a better time

[If no longer interested, thank them again for completing the survey and end the call.]

Intro B

I am calling concerning a study that [participant name] completed just recently and at that time indicated that he/she would like to participate in a phone interview as well. When would be a convenient time for me to call back to speak with [participant]?

CAM phone interview

[I. Welcome and introductions:]
Welcome and thank you for agreeing to participate in the CAM phone interviews. My name is [Author], and I will be conducting the interview today. My role as the interviewer is to direct the content and flow of the discussion and to make sure that we cover the main topics.

Before we begin, there are a couple important things I need to go over with you.

Taking part in the interview is completely voluntary. You may not benefit directly, but we do know that the information from this study will help us learn more about what CAM information needs cancer survivors have and how we can build tools to satisfy those needs. You may choose not to participate at all. If you decide to participate in the interview, you may stop at any time. If you decide not to participate, you will not be penalized in any way.

I also want to be sure you understand that this conversation will be recorded so we can analyze your responses later.

SC
Supplemental content to
J Med Libr Assoc. Jan;106[1]:dx.doi.org/10.5195/jmla.2018.200
www.jmla.mlanet.org
© Scarton, Del Fiol, Oakley-Girvan, Gibson, Logan, Workman 2018

Your answers will be kept strictly confidential and will not be shared with doctors or other health care providers. Records of participation in this research project will be kept confidential to the full extent permitted by law.

[II. Objective and agenda:]
First, let me explain the overall purpose of this interview. We are planning to develop a website that will allow patients with cancer to share their CAM successes and failures. The goal is to provide patients with a tool to understand what CAM therapies patients are finding effective and which have resulted in negative outcomes, as a means to meet the CAM information needs of cancer patients. For this interview, we are trying to determine what causes patients to have an interest in CAM, why they go outside the clinic for information, what they find, and how they evaluate that information.

[II. Procedures:]
Let's talk about how this interview will be conducted. If you don't understand something we are talking about, please feel free to ask questions at any time.

We will ask you to recall what first triggered your interest in CAM. Let me give you a few minutes to recall the situation.

[IV. Interview guidelines:]
Let's start with a few guidelines for todays' interview. Our interview will last approximately twenty minutes. Here are some basic guidelines for the interview:

- **First, there are no right or wrong answers.** This is VERY important. All of your thoughts and ideas are important to us. Only you understand your motivations toward CAM information seeking and how you felt about what you found.
- **Please speak up so that the tape recorder picks up your comments.**
- **All of the information collected today will be completely confidential.** Our discussion will be tape-recorded. All information from the taped interview will be transcribed, and all references to names eliminated. We can turn the tape recorder off at any time.
- **All of your thought processes will be very helpful.** Please feel free to add any thoughts pertaining to CAM information seeking or any gut feeling that might not even seem particularly relevant.

[Turn on tape recorder]

We appreciate the time you are generously giving to this important research study. Are there any questions before we begin? Okay, let's get started!

[Questions: 17 questions total]

[Incident identification]
Be aware that as I ask the questions in this interview, you may repeat yourself. This is OK. At times, this repetition can result in your recalling greater detail each time.

1. Please describe, as much as you can, your cancer history, from diagnosis through today and at each point, what first triggered your interest in CAM? What were your specific goals and objectives at the time? *[Goals and objectives]*
2. Where was the first place you went for information on CAM, and why was that your first choice?
3. Did you get the answers you needed at that time, or did you have to look elsewhere? What was it about that first avenue that resulted in unmet information needs and your going elsewhere?
4. Now let's focus on one specific CAM that you feel has been highly beneficial to your well-being and that you take regularly.

[Timeline]

Supplemental content to
J Med Libr Assoc. Jan;106[1]:dx.doi.org/10.5195/jmla.2018.200
www.jmla.mlanet.org
© Scarton, Del Fiol, Oakley-Girvan, Gibson, Logan, Workman 2018

For this CAM, can you put this into a brief timeline?
1. When did it start?
2. When did it end [you found the information you needed and started using it]?
3. What major events occurred between the start and end of the information-seeking process?

[Deepening]
Now we are going to review a specific CAM in more depth.
1. What caused you to seek out information on this particular product or how were you introduced to it?
2. What was the first decision you had to make?
    a. What information did you use when making this decision?
    b. Where did you get the information and from whom?
    c. Why did you use this information source? *[Information]*
3. What other options were considered or were available to you at that time, such as written materials, brochures, etc.? How were these options chosen or rejected? *[Options]*
4. Please explain some of the possible consequences of including this CAM and trusting the information you used in making that decision. Did you imagine the possible consequences of alternative options? *[Mental model]*
5. What led you to believe that you had made a good choice and would include this CAM in your treatment plan? How long did it take you to actually make the decision? Were physician preferences or preferences of other people involved in your decision? *[Decision making]*
6. Did you seek any guidance from other people such as other cancer survivors or from health professionals such as naturopathic doctors? From whom? What helped you to trust the guidance you received? *[Guidance]*
7. Was there anything about the CAM information-seeking and decision-making process that you found stressful? *[Difficult features]*
8. With all that you have learned from this information-seeking experience, if you had a health decision to address today and wanted to understand your options, where would you turn first for answers?

[What-if queries]
1. If a website were available where you could communicate with patients with the same cancer that you had and who were using [CAM], would you find it useful? *[Aid]*
2. Consider the tools available in social networking today such as communicating person-to-person, blogging, sharing links, and sending attachments, etc., what features of a site like this would you find essential for meeting your CAM information needs? *[Aid]*

[V. Wrap up]
Those are all the questions we have today. Is there anything else you would like to tell us about your CAM information seeking or use?

Thank you for your great input today/tonight. This will be very helpful in helping us understand patient information needs and building tools to meet those needs.
